# Chicago Medical Society

**Published:** 1872-10-01

**Authors:** 


### CHICAGO MEDICAL SOCIETY.

After the reading of the minutes of the previous meeting, Dr. Millard, the essayist, being called, presented a paper on Pulmonary Tuberculosis. Briefly reviewing the literature on the subject, the author called attention to the importance of recognizing the condition of the system which preceded the development of active morbid phenomena; and then, alluding to the various views that have been in vogue from time to time concerning the intimate nature of the primary deviation from health which occasioned or constituted the diathesis, he revived the idea that pulmonary consumption was intimately connected with, or dependent upon, a too rapid oxidation of the tissues. This was the central idea of the paper; and the greater part of its volume was formed by arguments adduced in its support. The author did not attempt to go further back, and explain the cause of the absorption of an undue quantity of oxygen; or the condition of system which preceded this manifestation. In his remarks on treatment, which were very brief, cod liver oil and iron were highly spoken of, but no allusion was made to such agents or measures as might be useful in diminishing the supply of the pernicious gas.

In the discussion that ensued, Dr. N. S. Davis, after alluding to the mortality of the disease, and to the observations of Prof. E. Andrews concerning the extent of its prevalence compared with other constitutional disorders, said that he had never regarded a narrow chest, or a chest encroached upon by tumors or spinal curvature, as rendering the individual peculiarly exempt from tuberculosis. He had yet to learn that a moist atmosphere, in which the proportion of oxygen was diminished, or imperfect ventilation, retarded the progress of the disorder. He had, on the contrary, been in the habit of advising consumptives to practice forcible inspirations; to exercise in such a manner as would promote expansion of the chest; to secure good ventilation; and to remove to parts in which the air was dry, and anything but deficient in oxygen. He thought tuberculosis had several modes of origin. The child of consumptive parents is peculiarly susceptible to the action of disturbing influences; there is in it an abnormal irritability of tissue which render it peculiarly alive to the action of the most trifling stimuli, natural as well as artificial. The circulation of such a child is excited; its pulse quick and frequent; its temperature unnaturally high; and it is acutely alive to atmospheric vicissitudes. Such individuals are necessarily prone to disease; and especially that of an inflammatory nature; and the inflammation is characterized by the abnormal multiplication of young or imperfectly formed cells. In such organisms, normal stimuli call forth exaggerated or perverted action; and the natural consequence is perverted nutrition. The tissues are morbidly impressed by the elements of the blood proper for their repair, and imperfectly assimilate them; while elements of a lower grade of vitality or of a less stimulant quality may be appropriated. Imperfect disintegration of tissues would result in essentially the same condition: particles imperfectly organized would be retained as constituents of living tissues, which in a condition of perfect health are further charged and excreted. Again, consumption not unfreqently occurs in individuals who have not inherited any tendency to it.

The Nails in Diagnosis of Paralysis.—Dr. S. Wier Mitchell, *Philadelphia Medical Times*, says that in cerebral palsies, whether from clot or embolus, there is an entire cessation of nail growth on the palsied side. Usually, when they begin to grow again, it is a sign that the power of movement will also improve in a few days. The rate of growth slowly increases, but it generally requires four or five months for such nails to produce an entire length from matrix to free edge. To study the change, I stain the nails of both sides with nitrate of silver or nitric acid. Staining is not, however, essential, except for comparison, because the line of arrested growth is marked by a deep groove, which for months may be seen as it passes down the nail, so that when accustomed to the rate of growths, the place of this furrow will enable an observer to guess pretty well at the date of the attack of paralysis. The palsy need not be complete to cause this arrest. It is found in cases involving either cerebral-motor palsy or sensori-motor paralysis, but, as yet, I do not know whether or not in the rare cases of pure sensorial palsies of cerebral origin it also exists, nor as yet have I any experience which enables me to say whether or not, in sudden spinal palsies, there is complete cessation of nail growth.

It seems to me possible that the nail growth may not be altered in the same degree by lesions of the cerebrum, cerebellum, pons, and corpus striatum; and I have some observations which appear to point hopefully to these facts of nail growth as a future means of aiding us to tell what parts of the brain have been attacked. Very recently one distinct, and, as I believe, most valuable practical contribution to diagnosis has come out of my observations. It is briefly this: In all sudden cerebral palsies, the nails cease to grow. In hysterical palsies of one limb, or of both, whether paraplegic or hemiplegic, the rate of nail growth is unaltered.—*Med. Record.*

Chloral as a Topical Application in Eczema.—A correspondent of the *Boston Med. and Surg. Journal* states that during the past year he has used in several cases of chronic eczema, with much satisfaction to his patients and himself, a solution of the hydrate of chloral as a topical remedy, in the proportion of one or two drachms to a pint of water, applied two or three times a day.—*Ibid.*

Death of Louis.—The celebrated French physician Louis has lately died, at the age of eighty-six.

